# Gramicidin, a Bactericidal Antibiotic, Is an Antiproliferative Agent for Ovarian Cancer Cells

**DOI:** 10.3390/medicina59122059

**Published:** 2023-11-22

**Authors:** Min Sung Choi, Chae Yeon Lee, Ji Hyeon Kim, Yul Min Lee, Sukmook Lee, Hyun Jung Kim, Kyun Heo

**Affiliations:** 1 Department of Biopharmaceutical Chemistry, Kookmin University, Seoul 02707, Republic of Korea; k1choi4@kookmin.ac.kr (M.S.C.); minyzoa@kookmin.ac.kr (Y.M.L.); lees2018@kookmin.ac.kr (S.L.); 2 Biopharmaceutical Chemistry Major, School of Applied Chemistry, Kookmin University, Seoul 02707, Republic of Korea; panda3364@kookmin.ac.kr (C.Y.L.); kjh0808@kookmin.ac.kr (J.H.K.); 3 Antibody Research Institute, Kookmin University, Seoul 02707, Republic of Korea

**Keywords:** gramicidin, drug repositioning, ovarian cancer, apoptosis

## Abstract

*Background and Objectives*: Gramicidin, a bactericidal antibiotic used in dermatology and ophthalmology, has recently garnered attention for its inhibitory actions against cancer cell growth. However, the effects of gramicidin on ovarian cancer cells and the underlying mechanisms are still poorly understood. We aimed to elucidate the anticancer efficacy of gramicidin against ovarian cancer cells. *Materials and Methods*: The anticancer effect of gramicidin was investigated through an in vitro experiment. We analyzed cell proliferation, DNA fragmentation, cell cycle arrest and apoptosis in ovarian cancer cells using WST-1 assay, terminal deoxynucleotidyl transferase dUTP nick and labeling (TUNEL), DNA agarose gel electrophoresis, flow cytometry and western blot. *Results*: Gramicidin treatment induces dose- and time-dependent decreases in OVCAR8, SKOV3, and A2780 ovarian cancer cell proliferation. TUNEL assay and DNA agarose gel electrophoresis showed that gramicidin caused DNA fragmentation in ovarian cancer cells. Flow cytometry demonstrated that gramicidin induced cell cycle arrest. Furthermore, we confirmed via Western blot that gramicidin triggered apoptosis in ovarian cancer cells. *Conclusions*: Our results strongly suggest that gramicidin exerts its inhibitory effect on cancer cell growth by triggering apoptosis. Conclusively, this study provides new insights into the previously unexplored anticancer properties of gramicidin against ovarian cancer cells.

## 1. Introduction

Ovarian cancer (OC) is one of the most lethal forms of gynecological malignancies, ranking as the eighth leading cause of cancer-related deaths among women globally [1]. In 2023, 19,710 new cases of OC and 13,270 deaths from OC are expected in the United States alone [2]. Most OC cases (90%) are epithelial ovarian cancer, and serous carcinoma is the predominant histotype. The lack of discernible early symptoms, the limited early detection methods, the diagnosis at advanced stages (70% of cases), the dearth of efficient screening techniques, and the intricate tumor heterogeneity contribute to the high mortality of patients with OC [1,3,4,5]. Following debulking surgery, the current therapies for OC include platinum-based chemotherapy, such as carboplatin, angiogenesis inhibitors, such as bevacizumab, and poly-ADP-ribose polymerase inhibitors (PARPi), such as olaparib. Despite advances in advanced OC treatment, 70–80% of patients who undergo first-line chemotherapy eventually experience disease recurrence. The targeted application of PARPi is effective in treating patients with OC with *breast cancer susceptibility gene* (*BRCA1/2)* mutations and certain homologous recombination deficiency-positive tumors due to the selectivity of PARPi for cells with homologous recombination defects. However, the prevalence of *BRCA1* or *BRCA2* mutations in patients with OC is relatively low, comprising only approximately 15% of the entire patient cohort. Intriguingly, a substantial proportion, from 40% to 70% of patients with *BRCA1/2*-mutated OC, do not respond favorably to PARPi [4,6,7]. Given these challenges, innovative and novel therapeutic approaches targeting OC are urgently needed to address this persistent unmet medical requirement and improve the outcomes for patients with OC.

The development of novel and potent anticancer therapeutics typically requires 10–15 years and a substantial financial commitment ranging from USD 1 to 1.5 billion. In response to these formidable challenges, drug repositioning (DR) has emerged as a prominent strategy over the past decade. DR is a methodology for identifying fresh target molecules or applications for existing drugs via modifications in formulation, dosage, or route of administration, thereby obviating the need for structural alterations. Candidates for DR include drugs with established safety profiles from human clinical trials, but the drugs may have encountered setbacks during phase 2 or 3 trials or were withdrawn from the market due to adverse effects. By capitalizing on the pre-established safety, pharmacodynamics, and pharmacokinetics profiles verified via clinical trials, DR can abbreviate the development timeline (3–12 years) and substantially reduce costs (50%–60% of novel drug development). Furthermore, drugs developed via DR have a 150% higher probability of entering the market compared to entirely new drugs [8,9,10,11].

Gramicidin, a pentadecapeptide antibiotic derived from *Bacillus brevis* (ATCC 8185) [12,13], functions as an ionophore, exerting its antimicrobial effects by disrupting ion potential and interfering with monovalent cation transport. This multifaceted channel activity confers antimicrobial effects against a spectrum of microorganisms, including Gram-positive bacteria, fungi, and protozoa [13,14]. Gramicidin also exhibits potent anticancer properties in various cancer cell types, including renal cell carcinoma and gastric cancer cells. Gramicidin inhibits cell growth and angiogenesis in renal cell carcinoma and proliferation and the cell cycle in gastric cancer cells [15,16,17]. However, the anticancer properties of gramicidin in OC cells and the underlying mechanisms are relatively unexplored.

In this study, we employed terminal deoxynucleotidyl transferase dUTP nick and labeling (TUNEL) assays, DNA agarose gel electrophoresis, Western blot analysis, and flow cytometry to elucidate the inhibitory effects of gramicidin on OC cell proliferation via apoptosis. Collectively, our results indicate that gramicidin is a promising therapeutic agent for the treatment of OC.

## 2. Materials and Methods

### 2.1. Cell Culture

The human epithelial ovarian adenocarcinoma cell lines SKOV3 and A2780 were purchased from the Korea Biotechnology Commercialization Center (Incheon, Republic of Korea). The OVCAR8 cell line was purchased from the American Type Culture Collection (Rockville, MD, USA). The cell lines were maintained in Roswell Park Memorial Institute medium (Thermo Fisher Scientific; Waltham, MA, USA), supplemented with 10% heat-inactivated fetal bovine serum (Gibco, Carlsbad, CA, USA), 100 U/mL penicillin, and 100 μg/mL streptomycin. All cells were cultured at 37 °C in a humidified incubator with 5% CO_2_.

### 2.2. Cell Proliferation Assay

Gramicidin was acquired from TargetMol (Boston, MA, USA). Cell proliferation was assessed using a WST-1 Cell Proliferation Assay Kit (#MK400, Takara, Kyoto, Japan) according to the manufacturer’s instructions. Briefly, 5 × 10^3^ OVCAR8 and A2780 cells and 2 × 10^3^ SKOV3 cells in 200 μL culture medium were seeded into 96-well tissue culture plates. After 24 h, the cells were treated with 0, 0.33, 1, or 3 μM gramicidin for 24, 48, and 72 h. Then, 20 μL of WST-1 reagent was added to each well. After incubation for 1 h at 37 °C, the absorbance of the formazan product was measured at 450 nm using a microplate reader (Synergy H1, BioTek, Winooski, VT, USA). IC_50_ values were determined by fitting the dose–response curves to a four-parameter variable slope sigmoidal dose–response model in GraphPad Prism 5.0 software (GraphPad Software Inc., San Diego, CA, USA).

### 2.3. TUNEL Assay

The TUNEL assay was performed using an in situ cell death detection kit and fluorescein, according to the manufacturer’s instructions (#11684795910, Roche, Meylan, France) with slight modifications. In brief, 4 × 10^3^ OVCAR8 and SKOV3 cells were seeded into 8-well chamber slides (#177402, Thermo Fisher Scientific) coated with 1 μg/mL of poly-L-lysine (Sigma-Aldrich, St. Louis, MO, USA). After 24 h, the cells were treated with 0, 0.1, 0.3, or 1 μM gramicidin for 48 h. After the gramicidin treatment, the cells were fixed in phosphate-buffered saline (PBS) with 4% paraformaldehyde for 10 min and permeabilized using 0.1% Triton X-100 in PBS for 3 min at room temperature. Cells were then resuspended in 50 μL TUNEL-reaction mixture for 1 h at room temperature (RT) and stained with Hoechst 33342 (#H3570, Invitrogen, Carlsbad, CA, USA) for 3 min. After washing twice with PBS for 3 min each, the mounting medium (#S3023, DAKO, Santa Clara, CA, USA) was added, and the slides were examined using a confocal laser scanning microscope (Leica, Wetzlar, Germany). The TUNEL intensity of individual cells was quantified using Image J software (version 1.54d) (NIH, Bethesda, MD, USA). The TUNEL fluorescence intensity was determined by dividing the mean intensity of apoptotic cells by the mean intensity of control cells.

### 2.4. DNA Agarose Gel Electrophoresis

OVCAR8 (4 × 10^5^) and SKOV3 (2 × 10^5^) cells were seeded in 6-well culture plates. After 24 h, cells were treated with 0, 0.1, or 0.3 μM gramicidin for 48 h. The cells were then lysed with TE (Tris-EDTA) buffer (10 mM Tris, 1 mM EDTA, and 0.05% Tween 20, pH 9.0; Bio-sesang, Yongin, Republic of Korea) containing 0.2% Triton X-100 (Sigma-Aldrich). After centrifugation at 1500× *g* for 5 min, the supernatant was collected and treated with 2 μg/mL RNase A (Merck, Burlington, MA, USA) for 30 min at 37 °C followed by treatment with 2 μg/mL proteinase K (Sigma-Aldrich) for 30 min at 50 °C. The DNA was precipitated by adding ammonium acetate (Bio-sesang) and absolute cold ethanol (Merck), followed by a 1 h incubation at −20 h °C. After centrifugation at 14,000× *g* rpm for 10 min, the supernatant was discarded, and the pellet was washed with 70% ethanol and resuspended with TE buffer. Finally, 1 μg of DNA from each sample was subjected to electrophoresis on a 1.5% agarose gel and stained with ethidium bromide (Thermo Fisher Scientific). The DNA fragments were visualized under ultraviolet illumination.

### 2.5. Cell Cycle Analysis Using Propidium Iodine (PI) Staining

Cell cycle progression was analyzed via flow cytometry. OVCAR8 cells (2 × 10^5^) and SKOV3 cells (2 × 10^5^) were seeded in 6-well culture plates and treated with 0.1, 0.3, or 1 μM gramicidin for 48 h. Subsequently, the cells were fixed, permeabilized using ice-cold ethanol at −20 °C overnight and washed twice with PBS. The cells were then subjected to staining with a DNA staining buffer (dH_2_O supplemented with 50 μg/mL propidium iodide (BD, San Jose, CA, USA) and 2 μg/mL RNase A (Sigma-Aldrich)) at 37 °C for 10 min, followed by an additional 20 min incubation at room temperature. Cellular DNA content was measured using flow cytometry. A minimum of 10,000 events were recorded and analyzed using a flow cytometer (SONY, N First St, CA, USA).

### 2.6. Western Blot Analysis

OVCAR8 and SKOV3 cells (2 × 10^5^) in 6-well culture plates were lysed with RIPA buffer (25 mM Tris-HCl pH 7.6, 150 mM NaCl, 1% NP-40, 1% sodium deoxycholate, 0.1% SDS; Thermo Fisher Scientific) and protein concentrations were determined using a BCA Protein Assay Kit (Thermo Fisher Scientific). OVCAR8 and SKOV3 protein extracts (10 μg per sample) were separated by 12% SDS-PAGE and transferred to nitrocellulose membranes (Amersham, Little Chalfont, UK). Membranes were blocked with 5% bovine serum albumin in Tris-buffered saline plus 0.1% (*v*/*v*) Tween 20 (TBST) for 1 h at RT and then incubated overnight with primary antibodies against caspase-3 (1:1000; #14220), cleaved caspase-3 (1:200; #9664), PARP (1:1000; #9542), cleaved PARP (1:1000; #5625), and β-actin (1:1000; #sc-47778) (Santa Cruz, Heidelberg, Germany) at 4 °C. After washing six times with TBST, membranes were incubated with horseradish peroxidase-conjugated secondary antibody for 1 h at RT. Membranes were then washed six times with TBST, and chemiluminescence was detected using enhanced chemiluminescence (ECL; Thermo Fisher Scientific), followed by imaging using Amersham ImageQuant 800 (Cytiva, Logan, UT, USA).

### 2.7. Statistical Analyses

All statistical analyses were performed using the GraphPad Prism 5.0 software (GraphPad Software Inc., San Diego, CA, USA). Statistical differences between groups were assessed using one-way analysis of variance (ANOVA) with Dunnett’s multiple comparison test. *p*-values < 0.05 were considered statistically significant (* *p* < 0.05, ** *p* < 0.01, *** *p* < 0.001).

## 3. Results

### 3.1. Gramicidin Inhibits the Proliferation of Ovarian Cancer Cells

**Figure 1:**
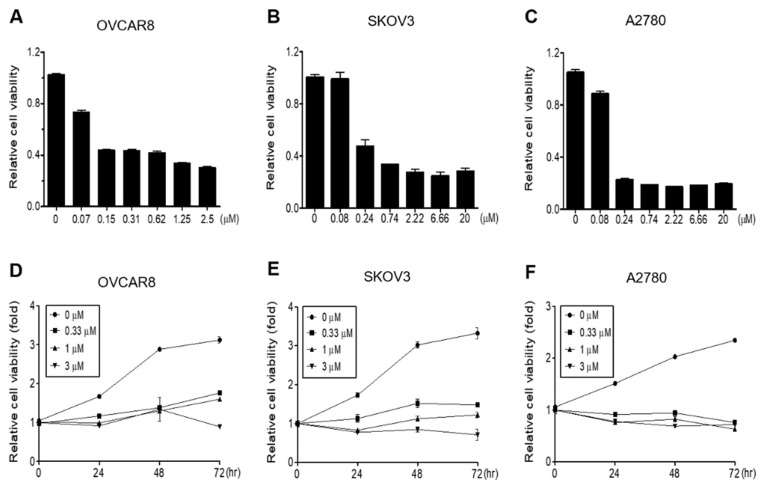
Gramicidin inhibited the proliferation of ovarian cancer (OC) cells. (**A**) OVCAR8, (**B**) SKOV3, and (**C**) A2780 cells were treated with the indicated concentrations of gramicidin for 72 h. (**D**) OVCAR8, (**E**) SKOV3, and (**F**) A2780 cells were treated with 0, 0.33, 1, or 3 μM gramicidin for 0, 24, 48, or 72 h. Relative cell proliferation rates were assessed using the WST-1 assay, which monitors mitochondrial succinate reductase activity at 450 nm. The data represent the mean ± SEM of duplicates from two independent experiments.

To investigate the inhibitory effects of gramicidin on proliferation, OVCAR8, A2780, and SKOV3 OC cells were treated with gramicidin, and proliferation was measured using WST-1 assays. A dose-dependent reduction in cell growth was observed across all three OC cell lines with IC_50_ values of 0.0763, 0.1856, and 0.1148 μM for OVCAR8, SKOV3, and A2780 cells, respectively (Figure 1A–C). As shown in Figure 1D–F, the impact of gramicidin on OC cell growth was also evaluated over time (0, 24, 48, and 72 h). Gramicidin inhibited OC cell proliferation in a time-dependent manner. Collectively, these findings indicate that gramicidin inhibits the growth of OC cells in a dose- and time-dependent manner.

### 3.2. Gramicidin Induces Apoptosis by Mediating DNA Fragmentation in Ovarian Cancer Cells

**Figure 2:**
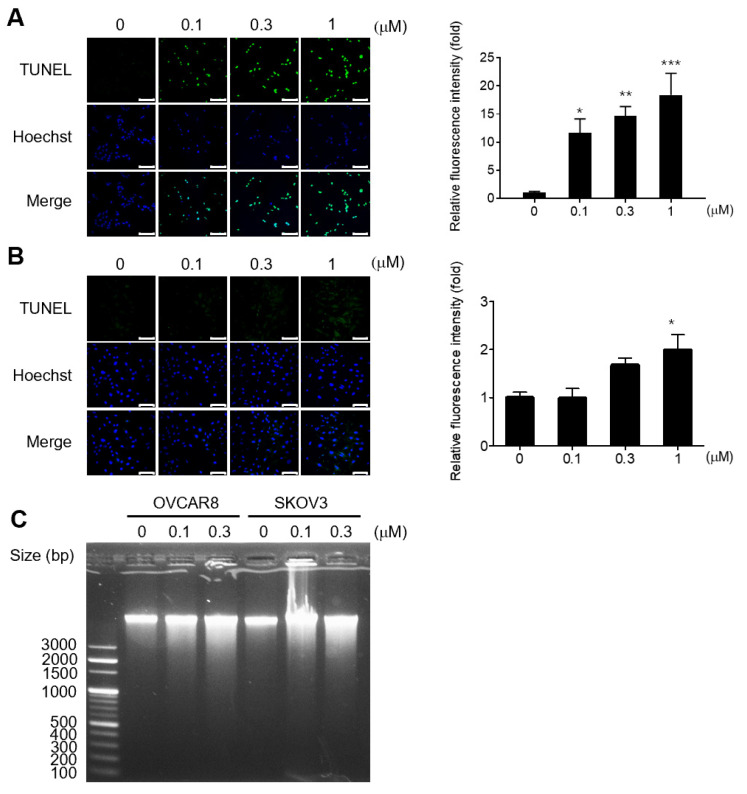
Gramicidin-induced apoptosis, as indicated by DNA fragmentation in OC cells. Apoptotic cells were detected by DNA fragmentation using the TUNEL assay and DNA agarose gel electrophoresis. (**A**) OVCAR8 and (**B**) SKOV3 were treated with 0, 0.1, 0.3, or 1 μM gramicidin for 48 h and imaged using confocal microscopy. TUNEL-positive nuclei are shown in green, and total nuclei stained with Hoechst 33,342 are presented in blue (left panel). Relative TUNEL intensities, normalized to the negative control, were quantified in five randomly selected microscopic fields (right panel). (**C**) OVCAR8 and SKOV3 were treated with 0, 0.1, or 0.3 μM of gramicidin for 48 h, and genomic DNA was isolated. DNA fragmentation was assessed by agarose gel electrophoresis with ethidium bromide staining. The data are presented as mean ± SEM of a minimum of three independent experiments. Statistical significance was calculated using one-way ANOVA (* *p* < 0.05, ** *p* < 0.01, and *** *p* < 0.001).

TUNEL assay was used to assess the apoptotic effects of gramicidin in OC cells. Gramicidin induced DNA fragmentation, indicating apoptosis, in OVCAR8 and SKOV3 cells in a dose-dependent manner when compared to the negative control (Figure 2A,B). DNA fragmentation agarose gel electrophoresis was performed to confirm the DNA fragmentation. Consistent with the TUNEL assay results, DNA fragmentation tended to increase with increasing gramicidin concentrations (Figure 2C).

**Figure 3:**
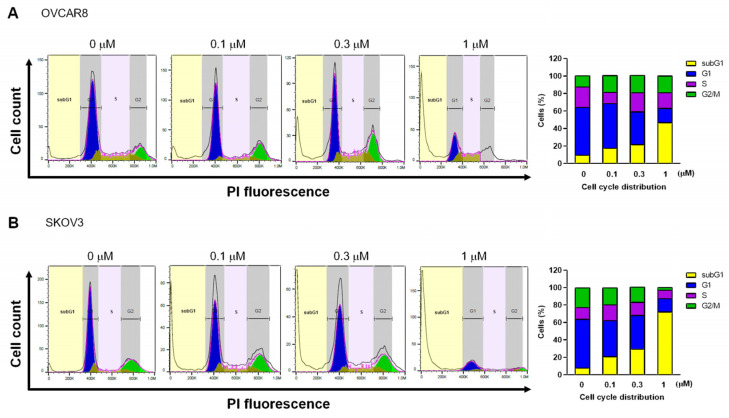
Gramicidin increased sub-G1 in OC cells. (**A**) OVCAR8 and (**B**) SKOV3 were treated with 0, 0.1, 0.3, or 1 μM of gramicidin for 48 h and subsequently stained with propidium iodide (PI) for cell cycle analysis using flow cytometry. The distribution of cells at different cell cycle phases was determined, and the percentages of cells in each phase relative to the total number of cells were calculated. The data are presented as mean ± SEM of three independent experiments.

A previous study reported that gramicidin induced G2/M cycle arrest in SGC-7901 cells [15]. Thus, we investigated the effects of gramicidin on the cell cycle distribution of OC cells using flow cytometry. Gramicidin treatment induced a significant increase in the sub-G1 phase in both OVCAR8 and SKOV3 cells, as shown in Figure 3. These results strongly imply that gramicidin influences DNA fragmentation and the cell cycle via apoptosis.

### 3.3. Gramicidin Mediates Apoptosis of Ovarian Cancer Cells via the Activation of Caspase-3

**Figure 4:**
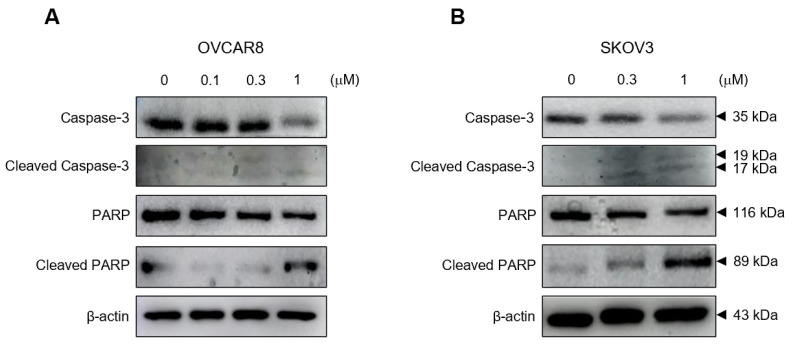
Gramicidin-induced cleavage of caspase-3 and PARP in OC cells. (**A**) OVCAR8 and (**B**) SKOV3 cells were treated with 0, 0.1, 0.3, or 1 μM of gramicidin for 24 h. Whole-cell lysates were analyzed via immunoblotting against caspase-3, cleaved caspase-3, PARP, cleaved PARP, or β-actin. The results represent a minimum of three independent experiments.

To determine the mechanisms underlying gramicidin-induced apoptosis in OC cells, the expression levels of the apoptosis-related proteins, caspase-3 and PARP, were determined with Western blot analysis. Gramicidin increased cleaved caspase-3 protein levels in both OVCAR8 and SKOV3 cells and substantially reduced the expression of caspase-3 protein. Cleaved PARP protein, a downstream target of caspase-3, was also increased, and the expression of PARP protein decreased (Figure 4). These data provide compelling evidence that gramicidin exerts its antitumor effect via the induction of apoptosis.

## 4. Discussion

In this study, we investigated the potential effectiveness of gramicidin for the treatment of OC via DR. Gramicidin inhibited the proliferation of OC cells, including the OVCAR8, SKOV3, and A2780 cell lines, in a dose- and time-dependent manner (Figure 1). TUNEL assay, cell cycle analysis, DNA agarose gel electrophoresis, and Western blot analysis using apoptosis-related antibodies in OVCAR8 and SKOV3 cell lines demonstrated that gramicidin induces apoptosis in OC cells. The apoptosis is characterized by DNA fragmentation and the cleavage of caspase-3 and PARP.

Gramicidin is a channel-forming polypeptide antibiotic composed of 15 L- and D-amino acids. The gramicidin monomer adopts a β-helix structure with an internal pore, and the monomers combine to form homodimers with β-helix pore structures that span the cell membrane. Dimerized gramicidin creates a pore through which water and monovalent cations can freely diffuse, resulting in Na^+^ influx and K^+^ efflux, membrane depolarization, osmotic swelling, and, ultimately, cell lysis [14,16,18]. Previous studies demonstrated the anticancer effects of gramicidin in various cancer cell types. For example, gramicidin inhibits the proliferation of cholangiocarcinoma cells (RBE and HuCCT1) [19]. Gramicidin also inhibited the proliferation of gastric cancer cells (SGC-7901), liver cancer cells (HepG2), and pancreatic cancer stem cells (BxPC3 and MIA PaCa-2) by inducing apoptosis [15,20,21]. Of note, the effects of gramicidin on OC cells have not been previously reported. This study represents the first investigation into the impact of gramicidin on OC cells, shedding light on its potential therapeutic significance in treating patients with OC.

Apoptosis is an energy-dependent programmed cell death process in multicellular organisms, leading to characteristic cellular changes via intrinsic and extrinsic pathways. Apoptotic cells exhibit morphological alterations, including cell shrinkage, blebbing, nuclear fragmentation, and chromatin condensation. Furthermore, apoptotic cells undergo biochemical modifications, including phosphatidylserine translocation from the inner to the outer membrane layer to facilitate phagocytosis by macrophages, protein degradation, DNA fragmentation, and the activation of caspases. Caspases, which can be classified into initiators and executioners, are cysteine proteases that cleave proteins after aspartic acid residues. The executioner caspases include caspase-3, which is activated via caspase cascades and plays a pivotal role in apoptosis. Caspase-3 cleaves various cellular proteins, cytoskeletal proteins, and DNA repair proteins [22,23]. In this study, cleaved caspase-3 and cleaved PARP increased in response to gramicidin treatment, suggesting that gramicidin induces the activation of caspase-3, which subsequently cleaves PARP—a known DNA repair protein—in OC cells (Figure 4). In apoptotic cells, chromosomal DNA is cleaved into oligonucleosomal-sized fragments by DNA fragmentation factors that are activated by cleaved caspase-3 in the late stages of apoptosis [24]. Our results demonstrate that gramicidin increases TUNEL fluorescent intensity (Figure 2), DNA fragmentation (Figure 2), and the percentage of cells in the sub-G1 phase (Figure 3) in OVCAR8 and SKOV3 cells. These results collectively indicate that DNA fragmentation is augmented in gramicidin-treated OC cells in a dose-dependent manner. Thus, our data strongly support the hypothesis that gramicidin induces apoptosis in OC cells.

The mechanism by which gramicidin induces apoptosis in cancer cells remains unknown. David et al. reported that gramicidin inhibited hypoxia-inducible factor 1α (HIF-1α) in renal cancer cells, consequently suppressing angiogenesis and tumor growth [25]. HIF-1α is an oxygen-sensitive subunit of HIF-1 and is expressed under hypoxia. Given that hypoxia is a common feature of many solid tumors, HIF-1α is frequently activated in cancer cells. Activated HIF-1α acts as a transcription factor, driving the expression of genes involved in various processes, such as survival, proliferation, metabolism, migration, and angiogenesis, in cancer cells [26,27]. Therefore, results indicate that gramicidin inhibits HIF-1α, in turn, inhibiting cell proliferation and inducing apoptosis in OC cells. However, further studies are required to investigate the anticancer effects of gramicidin via HIF-1α inhibition in OC cells.

In conclusion, our results support the DR of gramicidin as a potential therapeutic option for patients with OC. Gramicidin is a promising novel therapeutic agent for the treatment of OC.

## Data Availability

The data presented in this study are available upon request from the corresponding authors.
